# Dont's in Porcelain Inlays

**Published:** 1904-05

**Authors:** W. T. Reeves

**Affiliations:** Chicago, Ill.


### DONT'S IN PORCELAIN INLAYS.

By W. T. Reeves, D.D.S., Chicago, Ill.

Read before the Northern Ohio Dental Society.

Don't wait for a higher degree of perfection in inlay work before entering upon the work, because the time is at hand when every one should be prepared to do some of his filling with porcelain. There are numerous places where porcelain is the only material indicated and where today you should no more think of putting gold than you would think of putting amalgam. Besides, your patients are fast finding out that something better than gold can be put in their front teeth, something that harmonizes with the tooth, that is less taxation upon their nervous system to prepare the cavity, and altogether a desirable thing to have; they are asking for this kind of work, and if you are not prepared to do the work they go to some one who is. You cannot hold them much longer with the cry of "Fad, fad," that rolls off like "water from a duck's back." They have seen porcelain inlays in some friend's mouth, and know that they are doing all right. They size you up about right, that you can't do the work is the reason you cry "fad." Don't put it off any longer, but prepare yourself to do porcelain inlays.

Don't think that the esthetic is the only reason that porcelain should be used. That is one of the least of its many points of excellency. There is compatibility of material to tooth. Porcelain is the only permanent material we have that possesses this quality. Enough could be said on this one quality to make a paper by itself. There is permanency and preservation, two conditions that porcelain brings to the tooth to a greater extent than any other material.

Then there is ease of insertion, and that is a great feature in any operation—ease for both patient and operator. How many defective operations are allowed to pass because the operator is physically exhausted, or the patient is near nervous collapse, from the strain of a long, tedious gold operation? With porcelain all is different; patients hardly realize that they have been at the dentist's for two or three hours (until they get the bill), and leave the office with no dread of the future sitting. If it had been a gold operation, necessity is the only thing that would bring them back.

Don't make the mistake of thinking that porcelain is only indicated in a few places, and those of the simple order. Porcelain is only limited in range of application by the limitations of the operator. It is equally good for the young, the middle-aged and the old; everywhere and under all conditions, it is doing excellent service, and the more you use it the more you will find out and you will not be sorry.

Don't make up your opinion or judge by the failures you may have seen. If you decided what materials you would use and upon your *modus operandi* by the failures that come under your observation, would you be using gold today? I think not. You have no means of knowing what was the cause that brought about failure. It may have been gross incompetency on the part of the operator; he may have known nothing of the operative technic and still less of the material he was using. If, when he was a student, he had tried to accomplish an equal task with gold, with no better preparation, you would have laughed at him. Without thorough technic preparation, no better results should be expected than from the student with his first gold filling.

Don't think an outfit of bodies and a furnace is all there is to it—that when you have procured these you can begin on the next patient that comes in, and make an inlay, for you will be grievously disappointed.

Don't think that watching a clinic gives you all the insight you need of the technic, and that you can go to your office and do the same thing, for you will find that you are up against it,—that what looked so easy is quite another thing when you try it on a patient; that there are lots of little details that go to make up the sum total of success that have escaped you, and that you are stumped to know what to do; your patient will soon discover that you are stumbling along and will lose confidence in your ability to successfully accomplish the task. That would still further hamper you, and the result would be a failure. Your patients would realize that you had been experimenting on them. It is all right to experiment on some one else, but no one likes to be experimented on. Prepare yourself by the best technic instructions you can get; that puts you in possession of his experimentation and experience, and you begin at the point your instructor has attained. Go to the laboratory, and by careful dummy-work assimilate those instructions so that your patient will not know whether it is your first, tenth, or hundredth inlay. If it is not possible for you to make your start through this source, begin by reading and studying the literature up to date; try to sift the wheat from the chaff; adopt that which seems best to you; prove it by your work in the laboratory. Hold fast to the good and discard that which is inefficient in your hands, even if you have to begin all over again. This is a slower method, but what one gets by hard knocks usually sticks, and success follows earnest effort.

Don't think you can gain the knowledge of how to bake porcelain by hearsay or reading. Everyone must gain this through personal experience; the different stages of fusing can be shown and described, but experimentation is the only source of knowledge that will enable one to tell by observation just what degree of fusing has taken place. Porcelain may be a hard master, but, if understood, is a willing servant.

Don't pin your faith to what you have seen demonstrated at a table clinic. There are several methods that make awful nice table clinics,—interesting, entertaining, yes, fascinating; but if it was a practical inlay they were making for a patient, you would find the item of time was too expensive a feature for the busy man to seriously think of following.

Don't worship at golden altars or follow after false gods. Look on high (high-fusing porcelain) for your guiding star, and you will not have put your confidence in something that will fail you, and you will not be trailing behind.

Don't try to compound bodies. There are manufacturers who have made this a life study, and they can do this much better than you can ever hope to do. Learn the different bodies so that you can produce results. Study along this line will benefit yourself and the profession.

Don't try to make an inlay without first securing working space before beginning the operation. No more separation is needed than should be had for any other material, but it is absolutely necessary to have working space. You can't put in a flat inlay, but having proper space you will restore perfect contour, perfect contact points, and preserve the interproximal space better than with any other material. These three conditions will be appreciated by your patients, for it will put their teeth in a comfortable condition for masticating. You will be benefited and the standard of your work raised, for, finding how much your patients appreciate this change, you will separate for all other work and not consider it time wasted.

Don't set an inlay that is not a perfect fit at all points. With porcelain it is possible for you to know before it is too late to correct, whether there is any defect in adaptation, contour or color; if lacking in adaptation or contour, make another inlay. There is no physical strain upon yourself or patient to keep you from doing so. Many a defective piece of work is allowed to go through because the operator is not in condition to make a second gold filling. In making a second inlay, the effect upon the operator is two-fold: a failure overcome is a stimulus to renewed effort, and the moral tone of the operator has been raised.

Don't try to select the color your inlay is to be by taking the tooth-guide and selecting a facing that seems to match the tooth, and then working to that color, for you will be working to something once removed from the original, and no matter how well it might harmonize with the adjoining teeth if used as a crown, it would not match either tooth if a corner of the facing was cut off and placed against the tooth.

Don't take the shade-guide furnished with each outfit of bodies and try to select one that is a match for the tooth. The colors in the tooth vary from top to bottom, and you must have them in the same relative position in your inlay, in order to have inlay match tooth. Use the shade-guide, that by comparison with the tooth you can determine what colors you see in the tooth. The shade-guide gives you the color the body will be if you bake the same bulk and to a full glaze; you can get any shade of a given color by the thickness of the layer you bake.

Don't bake your inlay all of one body, even if you could make it in one baking, for your inlay would only match at one point and at the others would be off color, and then you would be up against what they call the "shadow problem."

Build in layers, bake in your colors strong enough that reflecting through an overlying or enamel layer gives you the colors you want; this will give you translucency and is the only way you can produce translucency; baking in layers, the strata break up light absorption and refraction and your inlay will look the same from all points of view. Baking the layers will prevent the cement showing through to the greatest extent possible.

Don't grind an inlay after it is set on any surface other than the grinding surface of molars and bicuspids, and the cutting edges of anterior teeth. All grinding that is necessary should be done before the matrix is removed, and then returned to the furnace and glazed. The glazed surface of porcelain is a protection to adjoining tooth surfaces.

Don't undercut your inlay with diamond disc or knife-edge stones; use hydrofluoric acid; that will remove the glaze and leave a roughened surface that cement will adhere to, and not take away from close adaptation.

Don't try to neutralize the acid. There is a fine, palpable powder as the result of the action of the acid, and this must be removed mechanically or it will interfere with the retention of the inlay; use water and a stiff bristle brush.

Don't set an inlay without putting on the rubber dam wherever it is possible to do so. Always have pressure either by wedge or ligature, and leave thirty minutes before removing dam; cement setting under pressure gives best results and prevents expansion making a noticeable joint.

Don't remove the excess cement until after you have removed the dam; after the saliva has bathed the tooth, the cement will come off easily with no danger of dislodging the inlay. ,

Don't try to remove the slight line of cement at the joint; let that wear away; it will wear away in five or six days.

Don't fail to tell your patient that the tooth has dried out under the rubber dam and is several shades lighter, and that it will be several hours before it is back to color, and that the inlay will look better a week hence than it does the first day.

Don't wait for others to discuss the subject or ask questions. A question that may seem trivial to you may be what lots of others would like to have answered.

---

DISCUSSION.

Dr. H. F. Harvey, Cleveland, O.: The first thing Dr. Reeves advises is not to wait until we have arrived at perfection in this work before undertaking to practice it. We should aim to come as near perfection as possible, and the only way to do this is to follow the advice he has given us in his paper; that is, get all the technical information we can and then go to the laboratory and practice. We cannot expect to arrive at perfection in the first operation or in a short time. This work certainly requires as much skill and care in every step of the work as anything else that we do. There are several reasons why this work should come into favor: first the condition of the patient; second, the condition of the teeth, and third, the esthetic side of the question. I hardly think the argument that it is easy for ourselves should have much weight.

The doctor says that we need no more space than we do for other fillings. I think this work requires more space than is needed for gold fillings. Another advantage is that you can correct imperfections. The lessening of the physical strain on the patient is certainly quite an advantage when you come to undertake a large filling.

---

Dr. S. B. Dewey, Cleveland, O.: Porcelain inlay work has come to stay. As the essayist says, we are not going to gain any knowledge or perfection if we wait for someone else to do the work. We must do it ourselves. I think in this matter of porcelain work not as many of us will become experts as with gold. It requires a person with a high conception of art to be able to select the colors and shade different colors to different teeth, and blend a variety of shades on the same tooth. We may not be artistic enough to produce the result that Dr. Reeves produces every day. It will take long years of practice before some of us can do this. Many teeth can be saved by this method and made useful and beautiful, which would otherwise disfigure the mouth of the wearer. I agree with the essayist that it is much easier in many cases for both patient and operator, and for those patients who cannot stand the long gold operations it is really a Godsend.

A man to be successful in this work must be able to give close attention to details. In reference to selecting colors: It helps me greatly not to match the tooth by the shade-guide, but look into the tooth and use the guide in comparison to what is seen in the tooth. Do not be too hasty in brushing off the excess of cement; it is better to leave it on for a while, although for a short time it may look unsightly.

---

Dr. W. G. Ebersole, Cleveland, O.: Porcelain inlay work has come to stay, and if we are going to make a success of it, we have got to work and work hard. There is no doubt of its being easy for the patient. I would advise the beginner to work a great deal on models and secure a certain degree of efficiency before attempting to put this work in the mouth. I am a disciple of porcelain work with high-fusing bodies.

---

Dr. L. L. Barber, Cleveland, O.: A matter of importance is the overcoming of thermal changes in the tooth. I believe there will be less cutting to do in the very sensitive part of the tooth. You have to remove the decay from the bottom of the cavity and that is about all you have to do. There are certain low-fusing bodies on the market that will stand and keep their color all right in some cases. If you have to grind the surface after your filling is made they will not keep their color, but you do not get the result that you do with high-fusing bodies which can be depended upon in all cases.

---

Dr. W. A. Siddall, Cleveland, O.: I have put in some fillings, and my trouble was not in trying to get them to come out, but I wished very much that they would stay in. I have learned also that when I start on a porcelain filling I do not know when I will be through, whether this week or next. Is it wise in corner teeth to make a step such as you would make for a gold filling, and also in bicuspid fillings where you can cut the matrix out? I have found that the cement will stick to the porcelain but will not stick to the tooth. What is the objection to slight undercuts?

---

Dr. Reeves, Chicago, Ill.: Dr. Harvey asked about space. He thought there were places where he could put gold fillings in with less space than necessary for an inlay. I think there are more places where you will get along with less space for inlays than otherwise. In molars and bicuspids you will surely get along with less space than you would need for gold fillings and have the full contour restored and get contact points that are simply perfection.

Dr. Dewey thinks there will not be as many expert operators in porcelain as in gold. There is where he makes a mistake. Porcelain work is in its infancy, we might say, having been introduced in the last five years, but I think that today you can find more expert inlay workers—ten to one—than you can find in the same grade of work with gold. You can't any of you come very near counting on the fingers of your two hands those operators whom you look up to as being good gold operators in this country. I think you can find twice as many as this who excel in porcelain inlay work in Chicago alone. I know a great many operators who are doing extremely good work in inlay, much better than they can do with gold, and they have only been working at it a year. This is something to be considered. You will come up so fast to the point of doing excellent work, if you start out earnestly, that it will surprise you how quickly you will be using it everywhere. The labial surfaces of anteriors are about the hardest you will have to deal with. Begin with the simple cavity for your first work. I believe any operator, wherever located, if he will give one-tenth of the time to the technic of porcelain inlay work that he gave to learning to put in gold fillings, he will have the best of results in this work. You can always be able to find the joint. You cannot slide across as you can with gold fillings. You will look into the tooth very carefully before discovering that there are inlays in the tooth.

Dr. Billings asked about corners. There are no corners where I would hesitate to place inlays. In any place where we can get cavity formation, porcelain can be used. The amount of undercutting you would do I do not think would give you any additional retention. The matrix must be adapted to the interior of the cavity and then the inlay set under pressure. A great many cases where you see the line at the margin, there has not been sufficient pressure. In regard to time, the inlay work will be accomplished, taking cavity for cavity, in a great deal less time than gold work. I have always used Harvard cement. There is a new cement now on the market which sets quicker than the original Harvard cement, but I have not used this on account of its producing a different mechanical action.

Dr. Price asked about platinum in small cavities. I use one one-thousandth. If the cavity is a pin-head cavity, use gold.

Dr. Price: Suppose the small cavity shows, would you enlarge it to the size of a No. 4 bur?

Dr. Reeves: I would. The question of cavity preparation I can hardly describe so that you can get a correct idea of it. The main thing is to remove all decay to good, sound, glistening enamel, get a good seating form for setting purposes so that after the matrix is taken off you could not tell which end is which, they are so near alike.

Someone asks, what about the price for porcelain inlay work as compared with the price for gold work? You should get from 25 per cent. to 30 per cent. more for porcelain inlay work than for gold.—*Summary.*

